# A Cortical Mechanism Linking Saliency Detection and Motor Reactivity in Rhesus Monkeys

**DOI:** 10.1523/JNEUROSCI.0422-23.2023

**Published:** 2024-01-03

**Authors:** Giacomo Novembre, Irene Lacal, Diego Benusiglio, Eros Quarta, Andrea Schito, Stefano Grasso, Ludovica Caratelli, Roberto Caminiti, Alexandra Battaglia Mayer, Gian Domenico Iannetti

**Affiliations:** ^1^Neuroscience of Perception & Action Lab, Italian Institute of Technology, Rome 00161, Italy; ^2^Department of Physiology and Pharmacology, University of Rome 00185, Sapienza, Italy; ^3^Cognitive Neuroscience Laboratory, German Primate Center – Leibniz-Institute for Primate Research, 37077 Göttingen, Germany; ^4^Neuroscience and Behaviour Laboratory, Italian Institute of Technology, Rome 00161, Italy; ^5^European Molecular Biology Laboratory (EMBL), Epigenetics and Neurobiology Unit, Rome 00015, Italy; ^6^Department of Neuroscience, Physiology and Pharmacology, University College London (UCL), London WC1E6BT, United Kingdom

**Keywords:** electroencephalography (EEG), event-related potentials (ERPs), force, local field potentials (LFPs), monkey, saliency

## Abstract

Sudden and surprising sensory events trigger neural processes that swiftly adjust behavior. To study the phylogenesis and the mechanism of this phenomenon, we trained two male rhesus monkeys to keep a cursor inside a visual target by exerting force on an isometric joystick. We examined the effect of surprising auditory stimuli on exerted force, scalp electroencephalographic (EEG) activity, and local field potentials (LFPs) recorded from the dorsolateral prefrontal cortex. Auditory stimuli elicited (1) a biphasic modulation of isometric force, a transient decrease followed by a corrective tonic increase, and (2) EEG and LFP deflections dominated by two large negative–positive waves (N70 and P130). The EEG potential was symmetrical and maximal at the scalp vertex, highly reminiscent of the human “vertex potential.” Electrocortical potentials and force were tightly coupled: the P130 amplitude predicted the magnitude of the corrective force increase, particularly in the LFPs recorded from deep rather than superficial cortical layers. These results disclose a phylogenetically preserved corticomotor mechanism supporting adaptive behavior in response to salient sensory events.

**Significance Statement** Survival in the natural world depends on an animal’s capacity to adapt ongoing behavior to abrupt unexpected events. To study the neural mechanisms underlying this capacity, we trained monkeys to apply constant force on a joystick while we recorded their brain activity from the scalp and the prefrontal cortex contralateral to the hand holding the joystick. Unexpected auditory stimuli elicited a biphasic force modulation: a transient reduction followed by a corrective adjustment. The same stimuli also elicited EEG and LFP responses, dominated by a biphasic wave that predicted the magnitude of the behavioral adjustment. These results disclose a phylogenetically preserved corticomotor mechanism supporting adaptive behavior in response to unexpected events.

## Introduction

Survival in the natural world depends heavily on an animal’s capacity to identify sudden threats or affordances and to quickly adapt ongoing behavior accordingly, with none or scarce influence of volition. We recently referred to this as reactive adaptive behavior (RAB): sudden sensory stimuli elicit swift involuntary behavioral responses that are, however, flexible on the basis of the current environmental context (Novembre and Iannetti, 2021).

There are multiple examples of RAB in the literature. One is corticomuscular resonance (CMR), which consists of a series of fast modulations of muscular activity in response to sudden and task-irrelevant sensory stimuli (Novembre et al., 2018, 2019 see also Somervail et al., 2021; Rangel et al., 2023). When humans exert a constant isometric force on a transducer held between the index finger and the thumb, such stimuli elicit an initial force decrease (*d1*, peaking ∼100 ms post-stimulus) followed by two consecutive force increases (*i1*, peaking at ∼250 ms; and *i2*, starting ∼300−350 ms and lasting for ∼2 s). These force modulations are tightly coupled, both on a trial-by-trial basis and across-subjects, to the large “vertex potential” elicited in the electroencephalogram (EEG) by the same stimuli evoking the CMR (Bancaud et al., 1953; Walter, 1964; Mouraux and Iannetti, 2009; Novembre et al., 2018). EEG responses like the vertex potential, as well as other responses such as the mismatch negativity and the P300, are believed to capture the violation of an internal model of the sensory environment (Picton, 1992; Näätänen et al., 2007; Luck, 2014). As such, the coupling between such EEG responses—classically associated to sensory systems—and motor output is intriguing. It suggests that updating a model of the sensory environment often and automatically triggers an action (or RAB), as it is indeed predicted by several models of saliency detection and orienting behavior (Sokolov, 1963; Neumann, 1990; Engbert and Kliegl, 2003; Menon and Uddin, 2010).

The CMR falls within the definition of RAB: it is elicited in an automatic and unconscious manner, that is, participants are unaware of producing a response, yet the force modulations is enhanced when the eliciting stimulus has high behavioral relevance (Novembre and Iannetti, 2021).

Thus, CMR responses, as well as RABs in general, are likely to be important for animal survival. As such, one would guess that these behavioral responses are well conserved phylogenetically. Yet, whether CMR is also observable in other species besides humans is unknown. Nevertheless, other RABs such as stimulus-locked responses (Corneil et al., 2004, 2008; Pruszynski et al., 2010; Goonetilleke et al., 2015), online motor corrections (Lee and Tatton, 1975; Battaglia-Mayer et al., 2013, 2014; Scott, 2016), or action stopping (Boehler et al., 2009; Schevernels et al., 2015; Wessel and Aron, 2017; Giarrocco et al., 2021) exist in both humans and nonhuman primates, suggesting that CMR might also be observable in nonhuman primates.

**Figure 1.**
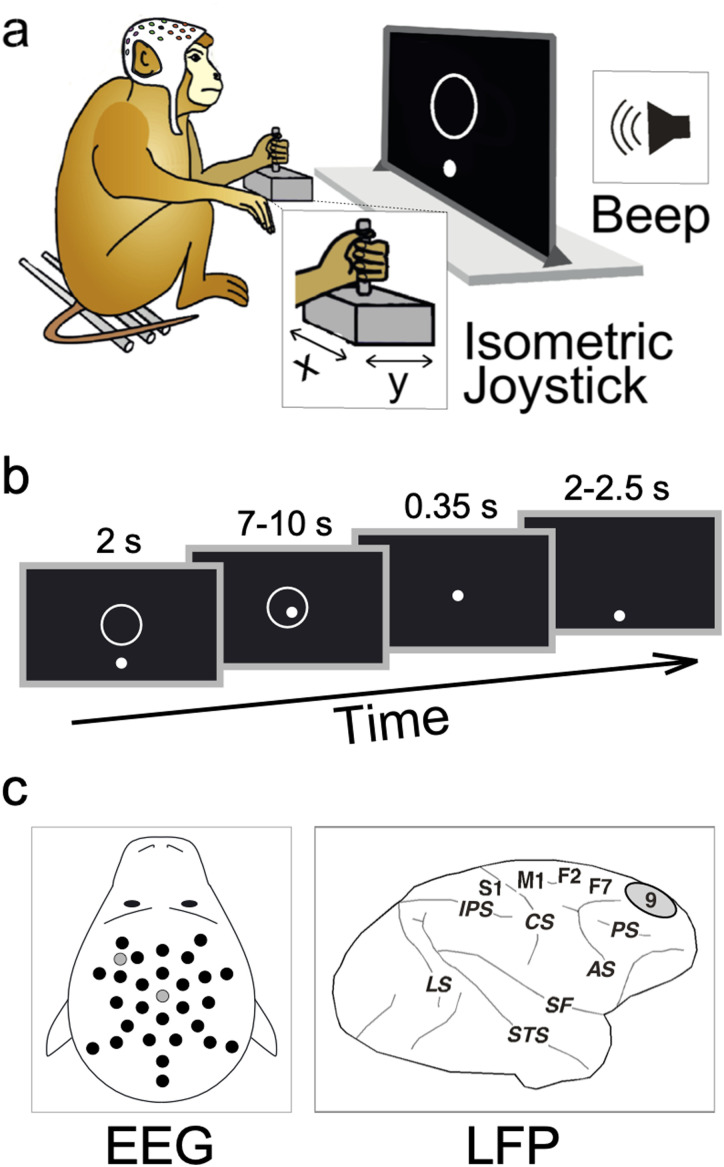
Experimental materials and methods. ***a***, Experimental paradigm. Two macaques were trained to exert force on an isometric joystick using the left hand. The force applied on the *x*- and *y*-axes of the joystick was used to control the position of a cursor (white dot) on a monitor. During a period of static force application, sudden task-irrelevant auditory stimuli were delivered through a beeper placed behind the monitor. ***b***, Task timeline. The task began with the presentation of a target (white circle) on the center of the screen. Monkeys had 2 s to bring the cursor inside the target and were required to hold the cursor there for a variable time interval (ranging between 7 and 10 s). In 33% of trials, auditory stimuli were unexpectedly delivered during this interval. If the cursor remained inside the target, the trial was considered successful, and a liquid reward was given. Trials were separated by a 2–2.5 s (jittered) interval (during which monkeys were not required to exert force and therefore the cursor was likely to be back to the start position). ***c***, EEG and LFP recording. In Experiment 1, we recorded EEG signals using 29 active electrodes (black dots) and 2 “zero-reference” electrodes (gray dots), mounted on custom-made EEG caps tailored to fit each animal’s head. In Experiment 2, LFPs were recorded from the right dorsolateral prefrontal cortex (Brodmann area 9), using a five-channel multiple-electrode array system for extracellular recording.

Therefore, the first aim of the current study was to investigate whether the CMR is present in nonhuman primates. To do so, across two experiments, we exploited a well-established behavioral task that requires rhesus monkeys (*Macaca mulatta*) to control the position of a cursor on a screen using an isometric joystick sensitive to handheld force (Fig. 1*a*) (Ferrari-Toniolo et al., 2015; Satta et al., 2017). Animals were trained to hold the cursor inside a central target, an action that implied the production of a small, constant force, while isolated fast-rising and task-irrelevant auditory stimuli were presented in a minority of the trials (Beep trials).

The second aim of this study was to investigate the neurophysiology of the CMR. In Experiment 1, based on the previous demonstration of a tight coupling between saliency-related vertex potentials and CMR in humans, we used 29 active electrodes to record electroencephalographic (EEG) activity in awake monkeys performing the task described above (Fig. 1). We examined event-related EEG potentials elicited by the salient stimuli and their relationship with the CMR. In Experiment 2, we repeated the above procedure recording intracortically local field potentials (LFPs) from the right dorsolateral prefrontal cortex (dlPFC, putatively Brodmann area 9, BA9)—a cortical area that has been shown to be involved in hand force control in both human (Ehrsson et al., 2000; Vaillancourt et al., 2007) and nonhuman primates (Badoud et al., 2017). Notably, BA9 lesioning leads to a bilateral impairment of fine hand force control, leaving general motor behavior intact (Badoud et al., 2017). The LFP recordings allowed us to compare the effect of responses measured at different cortical depths. This latter notion might shed light upon the specific circuits through which BA9 might contribute to the CMR.

## Materials and Methods

### Animals and surgical procedures

Two male rhesus monkeys (*M. mulatta*) participated to the experiments: monkey M (9 years old, 9.1 kg) and monkey T (9 years old, 9.4 kg). One headpost was mounted on the skull in each animal. In between Experiments 1 (EEG) and 2 (LFP), a circular chamber (diameter = 18 mm) was implanted for intracranial recording. The chamber was placed on the right hemisphere, centered at stereotaxic coordinates A + 35; L + 6 (Monkey 1) and A + 35; L + 7 (Monkey 2), in both cases corresponding to dorsolateral prefrontal cortex (specifically BA9). During the surgical procedures, the animals were pre-anesthetized with ketamine (10 mg/kg, i.m.) and then anesthetized with a mix of oxygen/isoflurane (1–3% to effect). Skull implants were performed under aseptic conditions. After surgery, the animals were allowed to recover for at least 7 days, while being treated with antibiotic and pain relievers, according to veterinary prescriptions. All efforts were made to minimize animals’ pain and distress. Animal care, housing, and surgical procedures were in agreement with European (EU Directive 63-2010) and Italian (DL. 26/2014) laws on the use of nonhuman primates in scientific research.

### Experimental setup

The experimental setup is illustrated in Figure 1*a*. Each monkey was placed in a soundproof chamber, seating on a primate chair in front of a 40-inch monitor (100 Hz, 800–600 resolution, 32-bit color depth; monitor–eye distance, 150 cm). Each animal was trained to control a white circular cursor by applying force on an isometric joystick with the left hand [consisting of a 1.5–6.5 cm metal cylinder mounted on top of a force transducer: FTS-Gamma (Calibration SI-32-2.5), ATI Industrial Automation]. The cursor [0.6 degrees of visual angle (DVA)] was displayed on a black screen. The force exerted on the transducer was sampled at 1 kHz on both the *x*- and *y*-axes, corresponding to hand force exerted toward the left/right (*x*-axis) and toward/away from the animal’s body (*y*-axis; Fig. 1*a*). Each animal faced the monitor from one out of two personalized primate chairs placed one next to the other. Consequently, monkey M had the monitor slightly on its right side (∼30° from the midline, i.e., at 1 o’clock) while monkey T slightly on its left side (approximately at 11 o’clock).

The force exerted on the transducer was used to control the position of the cursor on the monitor, so that a force of 20 N applied on the *y*-axis (away from the animal’s body) was necessary to hold the cursor in the central target (Fig. 1). Sudden and unexpected auditory stimuli were produced using a beeper placed behind the monitor, ∼160 cm away from the monkey’s head (Fig. 1*a*). Stimuli presentation and data sampling were controlled using the software package REX (Ferrari-Toniolo et al., 2019).

Both monkeys were required to use the left hand to perform the task, while the right arm was gently restrained. The joystick was controlled using the left hand because both monkeys appeared to prefer this configuration during the early stages of their training. We prevented the monkeys to reach their head with their arms by means of a 3D printed “safety box” (designed using Autodesk Fusion 360), that is, a nylon-12 surface that surrounded the animals’ neck and thus kept the EEG cap and electrodes away from the animals’ reach. Throughout the experiment, the monkey’s head was restrained using a titanium headpost.

### Behavioral task and paradigm

The task begun with the presentation of the target (an outlined white circle, 2 DVA in diameter) placed in the center of the screen, together with the visual cursor (a white dot, 3 DVA diameter), placed below the target when no force was exerted on the isometric joystick (Fig. 1*b*). The monkey was required to bring the cursor inside the target, by exerting a force of 20 N on the *y*-axis, i.e., away from the body (Fig. 1*a*). The animal had to enter the target within 2 s from trial onset (i.e., presentation of the target) and keep the cursor within the target until the end of the trial (i.e., disappearance of the target). Trial duration ranged from 7 to 10 s (rectangular distribution). If the monkey did not reach the target within 2 s from its appearance or did not hold the cursor inside it for the whole trial duration, the trial was aborted. Otherwise, the trial was considered successful, and the animal received 1.75 ml of liquid reward (Fig. 1*b*).

### Experimental paradigm

While holding the cursor within the target, monkeys experienced two types of trials. On one-third of the trials, a sudden auditory stimulus was presented (1 m distance, frequency 3.3 kHz, duration 50 ms). These trials are hereafter called “Beep trials”. The stimulus was always presented at least 3 s after the cursor had entered the target and not later than 3 s before the target disappearance. Within this time range, the timing of the stimulus was randomly assigned. On the remaining two-thirds of the trials, no auditory stimuli were presented, and monkeys were required to hold the cursor within the target for a comparable amount of time. These trials are hereafter called “No-Beep trials”. Beep and No-Beep trials were presented in a randomized order, within mini-blocks of 6 trials (2 Beep and 4 No-Beep trials), with the only caveat that no more than 2 Beep trials could be presented consecutively across successive mini-blocks.

### EEG equipment and montage (Experiment 1)

We recorded EEG using 29 active electrodes placed on the scalp (BioSemi Active-2 system). The data were sampled at 1,024 Hz. The electrodes were mounted on two custom-made caps (http://www.easycap.de), tailored to fit each animal’s head, according to the layout displayed on Figure 1*c*.

The BioSemi system replaces the ground electrodes with two electrodes named CMS (Common Mode Sense, active electrode) and DRL (Driven Right Leg, passive electrode). According to the system’s guidelines, CMS should (ideally) be placed in the center of the measuring electrodes, while DRL should be placed relatively away from them. While placing CMS, we also had to consider the position of the headpost, being approximately over Cz in monkey M and over Cpz in monkey T. Therefore, CMS was placed on Cz (in monkey T) and on Cpz (in monkey M). DRL was always placed on the frontal left side of the animal’s head (see the layout displayed in Fig. 1*c*; CMS and DRL are highlighted using gray dots).

### Intracortical recordings (Experiment 2)

Neural raw signals were recorded from area BA9, using a five-channel linear multiple-electrode array system for extracellular recording (Minimatrix 05, Thomas Recording). Interelectrode distance was 0.3 mm. Each electrode (platinum–tungsten fibers insulated with quartz 80 mm diameter, 0.8–2.5 MOhm impedance) was guided through the intact dura into the cortical tissue (one specific recording site per session) through a remote controller. The raw signal was amplified, digitized at 24 kHz, and transmitted through optical fibers to a digital signal processing unit (RA16PA-RX5-2, Tucker-Davis Technologies) where it was stored.

### Data analysis (Experiment 1)

In Experiment 1, we collected 327 successful Beep trials for monkey M (12 recording sessions, 27.25 ± 16.33 trials per session) and 365 successful Beep trials for monkey T (8 recording sessions, 45.62 ± 8.44 trials per session). These data were analyzed by applying the same pipeline (described hereafter) to the two datasets separately. This approach was preferred over the alternative “pooling” over the two datasets (Fries and Maris, 2021) because the latencies of the force responses observed in the two animals were not always overlapping in time (see below).

#### Force analysis

Continuous force data were low-pass filtered (35 Hz, Butterworth, third order) and then segmented into epochs of 3 s. For Beep trials, the epochs started 0.4 s prior to stimulus onset and ended 2.6 s following it. For No-Beep trials, equally long epochs were extracted relatively to randomly assigned time points comprised within the interval during which a stimulus could have been presented (i.e., at least 3 s after the cursor had entered into the target and not later than 3 s before the disappearance of the target). Force data comprised two channels *F*_x_ and *F*_y_ (associated with the force components exerted on the *x*- and *y*-axes of the transducer, respectively) and its magnitude *F* (which was computed using the following formula).

$$F=\sqrt{F_{x}^{2}+F_{y}^{2}}$$

Trials contaminated by artifacts (i.e., deviating > 4 SDs from the animal’s mean exerted force *F* across all trials) were excluded from further analyses (Novembre et al., 2018, 2019). The corresponding EEG time series were also excluded. These trials constituted 3.01% (monkey T) and 4.28% (monkey M) of the total number of trials. Epochs were baseline corrected using the −0.05 to 0 s prestimulus interval (Novembre et al., 2018, 2019). Beep and No-Beep trials were compared using point-by-point two-sample *t* tests.

#### EEG analysis

Continuous EEG data were bandpass filtered (1–35 Hz, Butterworth, third order) and then segmented into Beep and No-Beep epochs of 5 s (−1.4 to 3.6 s). Because the datasets contained several movement artifacts, data preprocessing was assisted by a validated algorithm for automatic artifact correction: artifact subspace reconstruction (ASR, threshold value = 5; Kothe and Makeig, 2013; Plechawska-Wojcik et al., 2018). ASR is an adaptive algorithm based on principal component analysis. It estimates clean portions of data to determine thresholds that are later used to reject large-variance components. The use of ASR was preferred over conventional “data cleaning” procedures because of its automaticity, implying lower computational time and lesser (potentially arbitrary) decision-making (Somervail et al., 2023). We note that we also compared the current results to those obtained following a traditional “data cleaning” procedure, which yielded similar results at the cost of several trials being rejected.

Following ASR, the EEG epochs were cropped to match the length of force epochs (i.e., −0.4 to 2.6 s). Noisy or faulty electrodes were interpolated by replacing their voltage with the average voltage of the neighboring electrodes. Data were re-referenced using a common average reference (Nunez and Srinivasan, 2006). Artifacts due to eyeblinks or eye movements were subtracted using a validated method based on an independent component analysis (Jung et al., 2000). In all datasets, independent components related to eye movements had a frontal scalp distribution. We also estimated the voltage at electrodes Cz and Cpz (used for CMS and for the headholder) by computing the average voltage of the neighboring electrodes. Finally, the EEG epochs were baseline corrected using the −0.2 to 0 s prestimulus interval. Beep and No-Beep trials were compared using point-by-point paired-samples *t* tests.

The trial-by-trial correlation between EEG and force (*F*) epochs was computed consistently with our previous work (Novembre et al., 2018, 2019). We first smoothed the signals using a moving average (sliding window = 20 ms). The signals were then resampled to 250 Hz to reduce computation time. Finally, the trial-by-trial correlation coefficient (Spearman’s *r*) was computed between EEG amplitude and force magnitude, for all possible pairs of time points between the interval −50 to 400 ms of the EEG time course (i.e., the interval encompassing all EEG modulations) and the interval −50 to 2,000 ms of the force time course (i.e., the interval encompassing all force modulations). This resulted in 29 correlation matrixes (one for each EEG electrode). Significant correlations were thresholded by extracting clusters encompassing at least two consecutive significant time points (*p* < 0.05) associated to at least two neighboring electrodes.

### Data analysis (Experiment 2)

In Experiment 2, we collected 393 successful Beep trials for monkey M (28 recording sessions, 14.04 ± 4.05 trials per session) and 339 successful Beep trials for monkey T (25 recording sessions, 13.56 ± 1.90 trials per session).

Behavioral data from Experiment 2 were analyzed by applying the same pipeline described for Experiment 1. Trials contaminated by artifacts (i.e., deviating >4 SDs from the animal’s mean exerted force *F* across all trials) were excluded from further analyses. The corresponding LFP time series were also excluded. These trials constituted 3.20% (monkey M) and 4.78% (monkey T) of the total number of trials.

Continuous extracellular LFP data were bandpass filtered (1–35 Hz, Butterworth, third order), polarity-inverted (for comparability with the EEG signal), and then segmented into Beep and No-Beep epochs of 5 s (−1.4 to 3.6 s). LFP data were recorded from five electrodes, each with a single recording site. Within each recording session, a variable number of electrodes failed to penetrate the dura and did not reach the target cortical depth. These electrodes were considered “non active,” and their corresponding LFP time series were excluded from the analyses [69 out of 140 (49.29%) for monkey M and 15 out 125 (12%) for monkey T]. The remaining “active” electrodes were classified as “superficial” or “deep” by applying a median split on the cortical depth from which recordings were taken.

The trial-by-trial correlation between LFP and force epochs was computed as in Experiment 1. Correlation matrixes were calculated by pooling all “active” electrodes together or by pooling “superficial” or “deep” electrodes separately. Significant correlations were thresholded for significant time intervals (*p* < 0.05).

## Results

### Stimulus-induced force modulations (Experiment 1)

**Figure 2.**
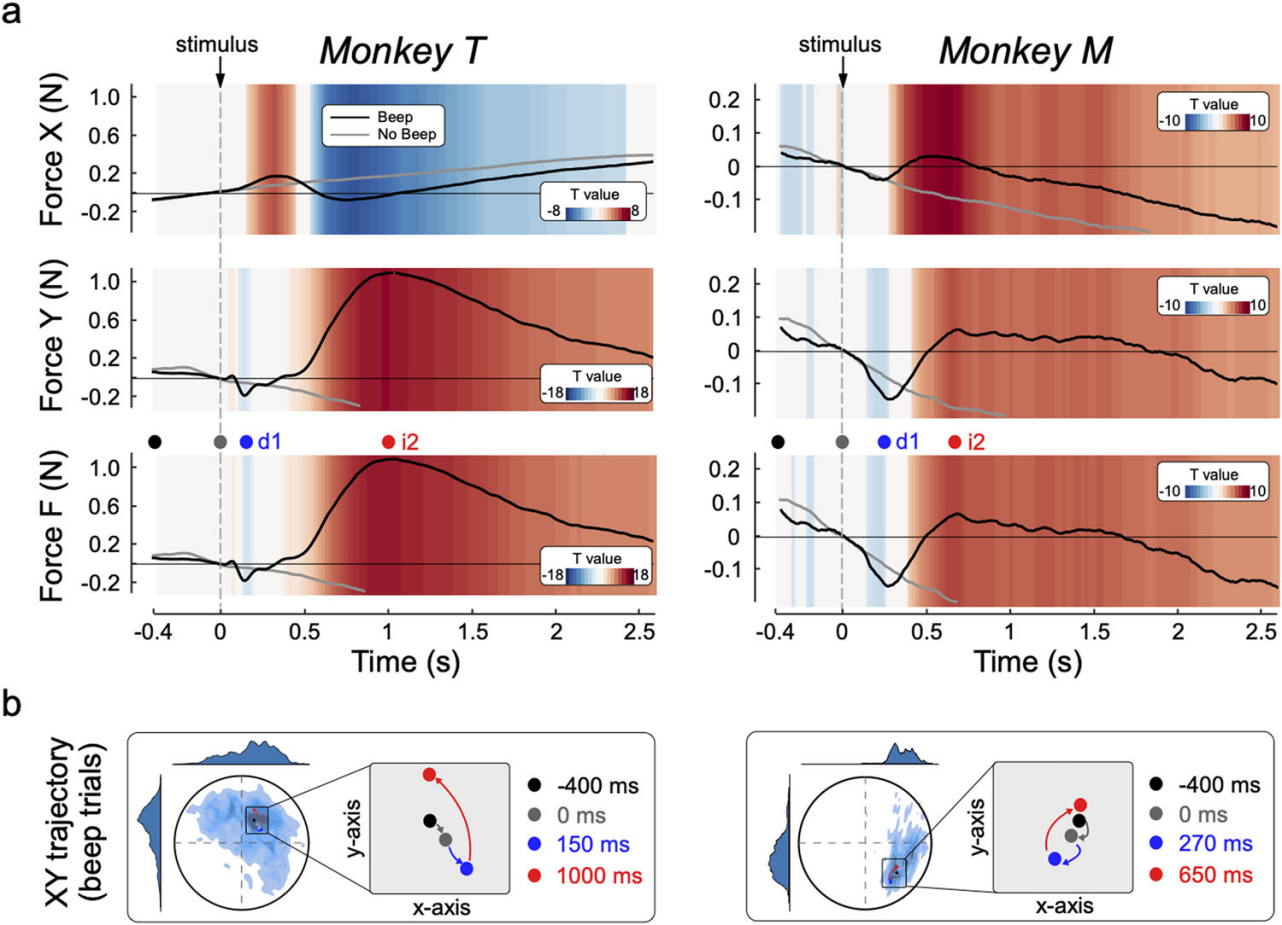
Stimulus-induced force modulations. ***a***, Stimulus-induced modulations of force magnitude over the *x*-axis (first row) and *y*-axis (second row). A composite index of force (*F*), representing the overall force magnitude regardless of its *x*–*y* directionality, is displayed in the third row. The colored background represents *t* values yielded after comparing Beep (black line) and No-Beep (gray line) trials. ***b***, Illustrative representation of the position of the cursor (dot) with respect to the target (circle) over time, at four different time points: baseline onset (black), stimulus presentation (gray), peak of force decrease (blue), and peak of force increase (red). The density maps represent all positions held by the dot over the course of all trials.

In both monkeys, auditory stimuli elicited a consistent biphasic modulation of force magnitude (*F*; Fig. 2*a*, third row): an initial force decrease was followed by a force increase. This pattern was strongly evocative of that previously observed in humans (Novembre et al., 2018, 2019), even though the latency of the current modulations was somehow inconsistent across animals and species, as we discuss below in more detail. Notably, when considering behavioral responses, a certain degree of both interindividual and interspecies difference is to be expected, consequent to the presence of unique individual strategies and perceptual–motor styles (Vidal and Lacquaniti, 2021).

The *t* tests comparing the exerted force across Beep and No-Beep trials (i.e., trials during which there was no auditory stimulus; see Materials and Methods) confirmed the across-trial consistency of the biphasic modulation of force magnitude, in each animal. To assist interpretability of these modulations with respect to their human equivalents, the initial force decrease and the following increase will be hereafter referred to as *d1* and *i2*, respectively.

The latency of the force modulation, particularly the initial *d1*, was different across animals. In monkey T, *d1* peaked at ∼150 ms, while in monkey M it peaked at ∼270 ms post-stimulus. In contrast, the subsequent *i2* was more similar across animals: it began ∼400–450 ms post-stimulus and lasted nearly the whole trial duration. Notably, and paralleling human observations (Novembre et al., 2018, 2019), in both animals *d1* was more transient than *i2*.

Examining the simultaneous modulations of force separately on the *x*- and *y*-axes (Fig. 2*a*, first and second row), we reconstructed the average cursor trajectory before and after the presentation of the auditory stimulus (Fig. 2*b*). In both monkeys, prior to stimulus presentation, the cursor slowly drifted toward the bottom of the screen (Fig. 2*b*, black arrow). Bearing in mind that a force resulting in an upward movement on the *y*-axis had to be exerted to keep the cursor inside the target, this observation is consistent with the well-known fatigue effect in isometric force tasks [which we and others also observed in humans (Nazir et al., 2017; Novembre et al., 2018)]. Immediately after stimulus onset, the first force decrease (*d1*) resulted in a transient enhancement of the above-described prestimulus drift (Fig. 2*b*, blue arrow). The subsequent force rebound (*i2*) moved the cursor in the opposite direction, bringing it above the prestimulus position (Fig. 2*b*, red arrow).

Comparing the direction of these motion trajectories across monkeys, we noticed that they were consistent along the vertical *y*-axis, but somehow different along the horizontal *x*-axis: in monkey T, the cursor drifted toward the right side of the monitor, while in monkey M it drifted to the left. This difference is possibly explained by the different position of each monkey relative to the monitor (slightly on the right side of monkey M and on the left side of monkey T; see Materials and Methods, Experimental setup). Thus, the different drifting along the *x*-axis might be trivially explained by the different hand and arm posture adopted by the two animals.

### Stimulus-induced EEG modulations (Experiment 1)

**Figure 3.**
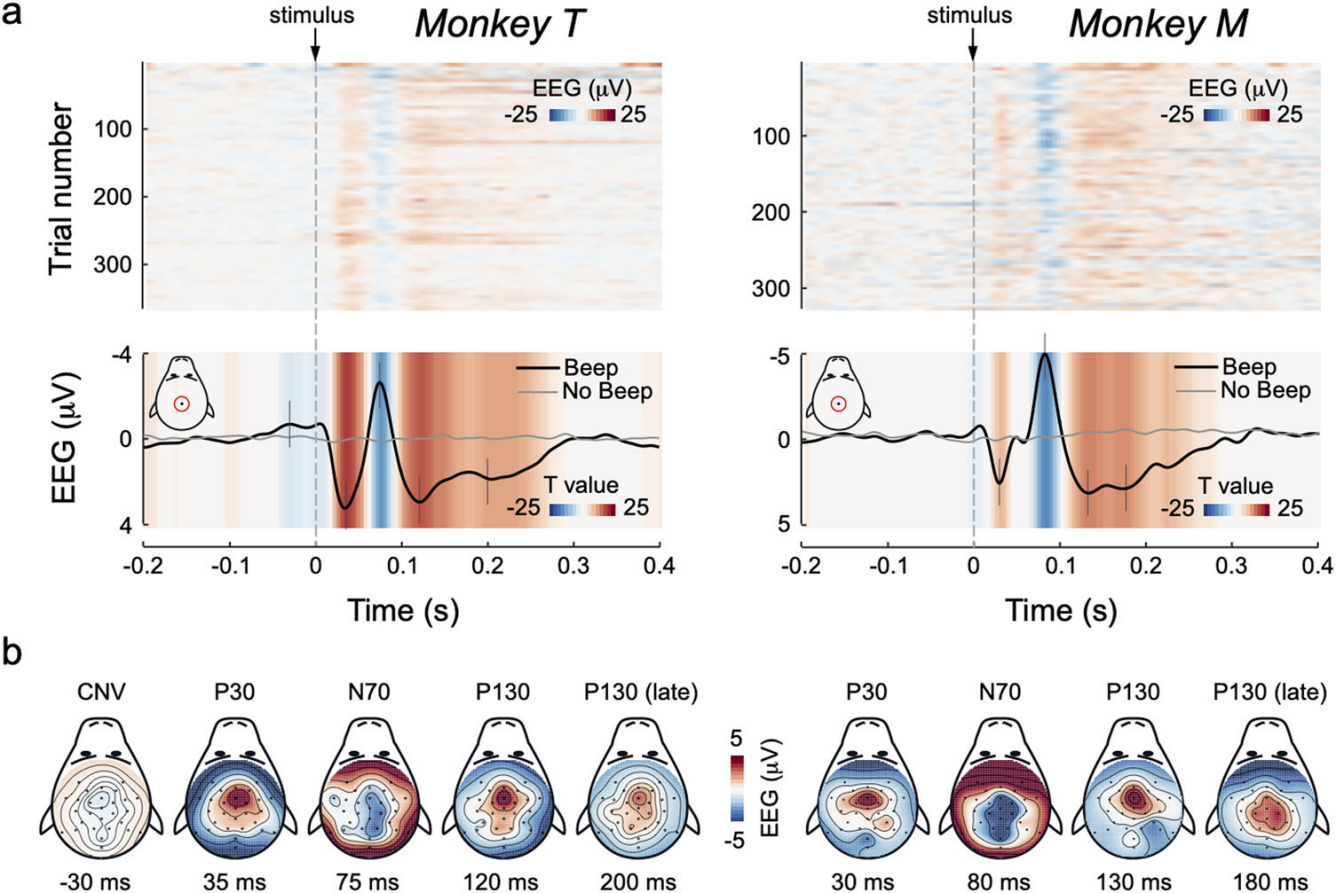
Stimulus-induced EEG modulations. ***a***, Top, Single-trial modulations (at electrode Cz). Trials are sorted by their order of occurrence. The colored background represents amplitude. Bottom, Across-trial averages of EEG modulations (at electrode Cz). The colored background represents *t* values yielded after comparing Beep (black line) and No-Beep (gray line) trials. ***b***, EEG topographies of the main modulations. Time points of each topography are marked with vertical gray lines crossing the EEG average waveform (shown in ***a***, bottom).

The EEG modulation elicited by the auditory stimuli is displayed in Figure 3. The modulation of EEG voltage consisted of a triphasic pattern including an early positivity (P30), a negativity (N70), and a second longer lasting positivity (P130). The negative-positive N70 and P130 complex constitutes the well-known vertex potential that can be measured in human and nonhuman primates (Bancaud et al., 1953; Neville and Foote, 1984; Pineda et al., 1989; Mouraux and Iannetti, 2009; Gil-Da-Costa et al., 2013; Milne et al., 2016).

Both latencies and topographies of these EEG modulations were remarkably consistent across animals. Specifically, the P30, which had central and frontal distribution, peaked at 35 and 30 ms post-stimulus in monkeys T and M, respectively. The N70 had broader and more posterior distribution over the scalp, and it peaked at 75 and 80 ms in monkeys T and M, respectively. Finally, the early part of the P130 exhibited a centrofrontal topography, peaking at 120 and 130 ms in monkeys T and M, respectively. Notably, the P130 lasted longer than the previous P30 and N70, and its initial frontal topography changed slightly throughout time, to become more widespread and centrally distributed ∼180–200 ms post-stimulus, particularly in monkey M (Fig. 3*b*). The *t* tests comparing the EEG voltage across Beep and No-Beep trials confirmed the high across-trial consistency of all the described components (Fig. 3*a*, bottom).

Notably, monkey T exhibited a mild slow-rising negativity anticipating the stimulus. This component is most likely a contingent negative variation (CNV; Walter et al., 1964; Borda, 1970).

### Trial-by-trial correlation between force and EEG modulations (Experiment 1)

**Figure 4.**
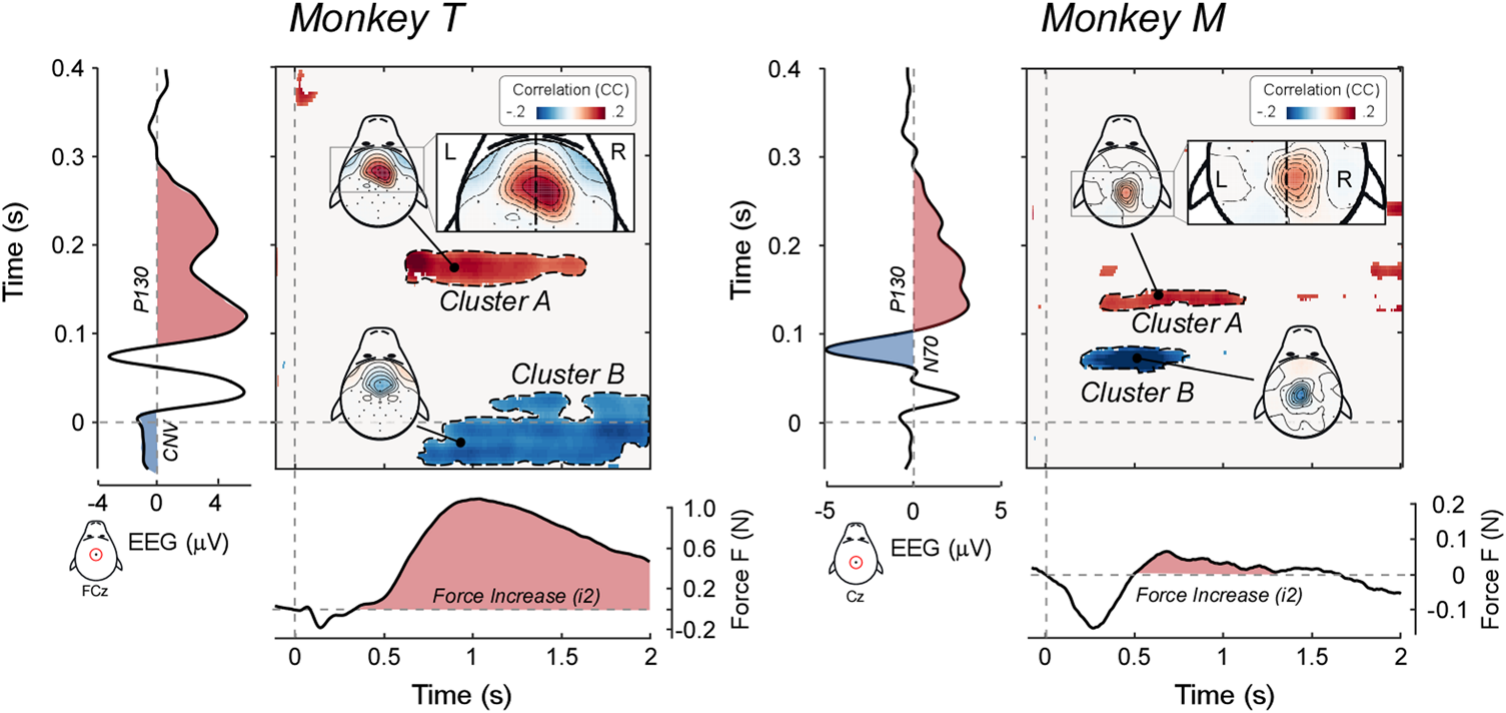
Trial-by-trial correlations between stimulus-induced force and EEG modulations. Bidimensional plots represent the significant trial-by-trial correlation coefficients (cluster-corrected Spearman’s *r*) between EEG and force, for all possible pairs of time points, at electrode Cz. The topographies of the main correlation clusters are also plotted. The EEG time series (plotted vertically) and the force time series (plotted horizontally) are shown to assist interpretability of the correlations. Note that the correlation between the EEG positive wave (P130) and the force increase (*i2*) is slightly lateralized toward the right scalp regions, that is, contralateral to the arm that exerted the force.

The trial-by-trial correlation between force and EEG modulations revealed several interesting relationships, which are outlined in Figure 4.

First, both monkeys exhibited a robust correlation between the P130 EEG wave and the force increase *i2* (Fig. 4, cluster A). This implies that trials in which the P130 had large amplitude were also associated with a large force increase. It is also important to examine *where* across the scalp this correlation occurred [i.e., where trial-by-trial fluctuations of EEG amplitude were more strongly coupled with fluctuations of *i2* magnitude (Novembre et al., 2018)]. In both animals, this correlation was stronger over the right hemisphere, that is, contralaterally to the (left) hand exerting the force (Fig. 4, inset). Remarkably, both the correlation between the positive vertex potential and the *i2* and the topography of such correlation were similar to what we previously observed in humans (Novembre et al., 2018, 2019).

We also observed two additional relationships between EEG and force modulations that, however, were not consistent across the two animals (Fig. 4, cluster B). First, in monkey M, the amplitude of the N70 correlated negatively with the magnitude of the force increase following *d1* (i.e., with the ascending branch of *d1* and the initial part of *i2*)—another result that parallels what we observed in humans (Novembre et al., 2018). Second, in monkey T, the amplitude of the CNV correlated negatively with the magnitude of *i2*.

### Trial-by-trial correlation between force and LFP modulations

**Figure 5.**
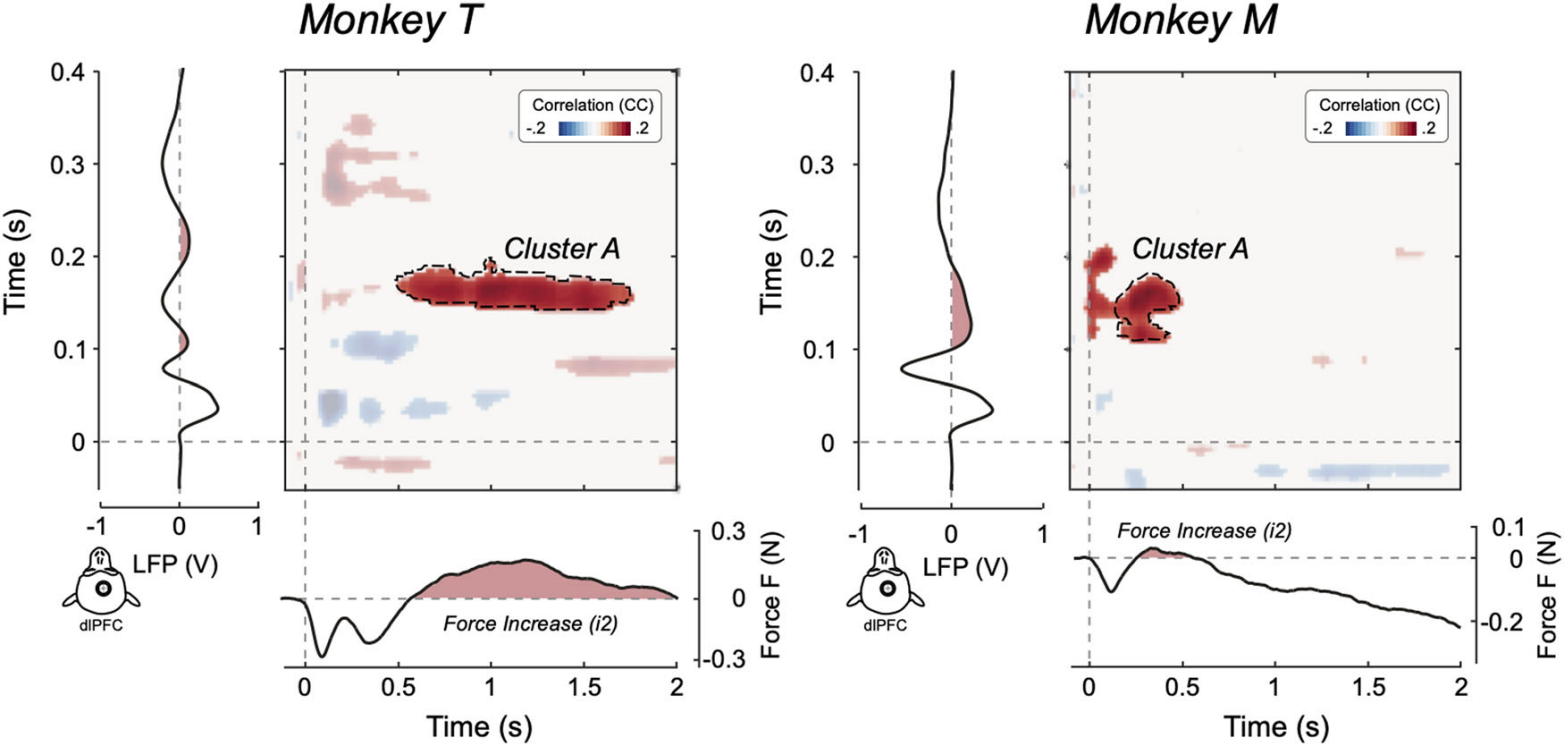
Trial-by-trial correlations between stimulus-induced force and LFP modulations (recorded from the dlPFC). The bidimensional plots represent the significant trial-by-trial correlation coefficients (cluster-corrected Spearman’s *r*) between LFP and force, for all possible pairs of time points (pooling all “active” electrodes). The LFP time series (plotted vertically) and the force time series (plotted horizontally) are shown to assist interpretability of the correlations. The correlation between the LFP positive wave (equivalent to the EEG P130) and the force increase (*i2*) is highlighted.

Experiment 2 revealed a pattern of force modulation broadly similar to the one observed in Experiment 1 (compare Figs. 2, 5). In both monkeys, auditory stimuli elicited modulations of the overall force magnitude (*F*) in a biphasic pattern composed of an initial force decrease (*d1*) followed by a force increase (*i2*). In monkey M, *d1* peaked at 148 ms post-stimulus, while *i2* peaked at 359 ms post-stimulus. In monkey T, *d1* showed a double peak (at 163 and 409 ms post-stimulus), due to an additional force increase peaking at 281 ms post-stimulus. The late force increase *i2* started ∼400 ms post-stimulus and peaked > 1 s post-stimulus. The morphology of these force responses, specifically that of the *i2*, was comparable to that described above (Figs. 2, 5).

The auditory stimuli also elicited LFP modulations markedly similar to the EEG modulations described above (compare Experiments 1 and 2; Figs. 3, 5). Specifically, these modulations entailed a triphasic pattern consisting of an early positivity (36 ms post-stimulus in both monkeys M and T), a negativity (78–79 ms in both monkeys M and T), and a second longer lasting positivity. In monkey M, this last positivity was very similar to what observed in Experiment 1 and peaked at 127 ms post-stimulus. In monkey T, this positive component appeared to be split into two halves (peaking at 106 and 215 ms post-stimulus, respectively), due to an additional negative deflection peaking at 151 ms post-stimulus. Looking more closely to the results from Experiment 1, this negativity embedded within the last P wave was also present in the EEG data (Figs. 3, 4, left), although less clearly than in the LFP data (Fig. 5).

**Figure 6.**
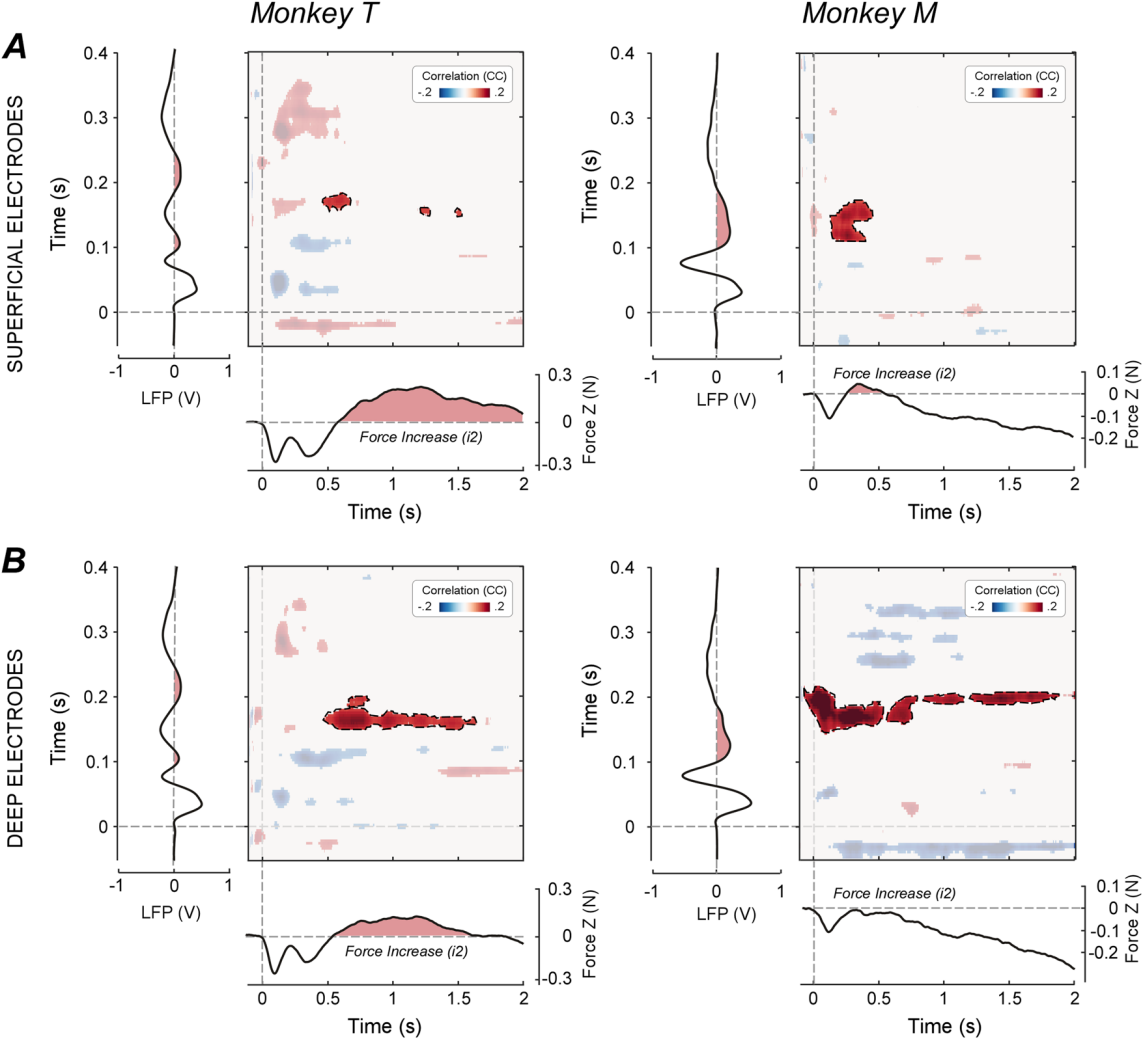
Trial-by-trial correlations between stimulus-induced force and LFP modulations at different cortical depths. The bidimensional plots represent the significant trial-by-trial correlation coefficients (cluster-corrected Spearman’s *r*) between LFP and force for all possible pairs of time points, for either superficial (***A***) or deep (***B***) recording sites. The LFP time series (plotted vertically) and the force time series (plotted horizontally) are shown to assist interpretability of the correlations. The correlation between the LFP positive wave (equivalent to the EEG P130) and the force increase (*i2*) is highlighted. Note the clearer LFP–force correlations in deep recording sites.

Most compellingly, the correlation between LFP and force data was extremely similar to that observed between EEG and force (compare Figs. 4, 5). Specifically, the late positivity evoked by the auditory stimulus correlated, on a trial-by-trial level, with the late force increase *i2*, in both animals (Fig. 5, cluster A). When we looked at this correlation as a function of cortical depth, that is, considering selectively deep and superficial recording sites, we found that the correlation between LFP and force was clearer for deep electrodes (Fig. 6).

## Discussion

In this study we investigated (1) whether the CMR—a multiphasic modulation of isometric force elicited by salient sensory stimuli—is present in nonhuman primates and (2) its neural correlates. In the next sections we compare the CMR observed in monkeys and humans and describe the neural responses elicited by the stimuli causing the CMR in monkeys, with particular emphasis on their tight coupling.

### Force modulations: CMR in rhesus monkeys?

**Figure 7.**
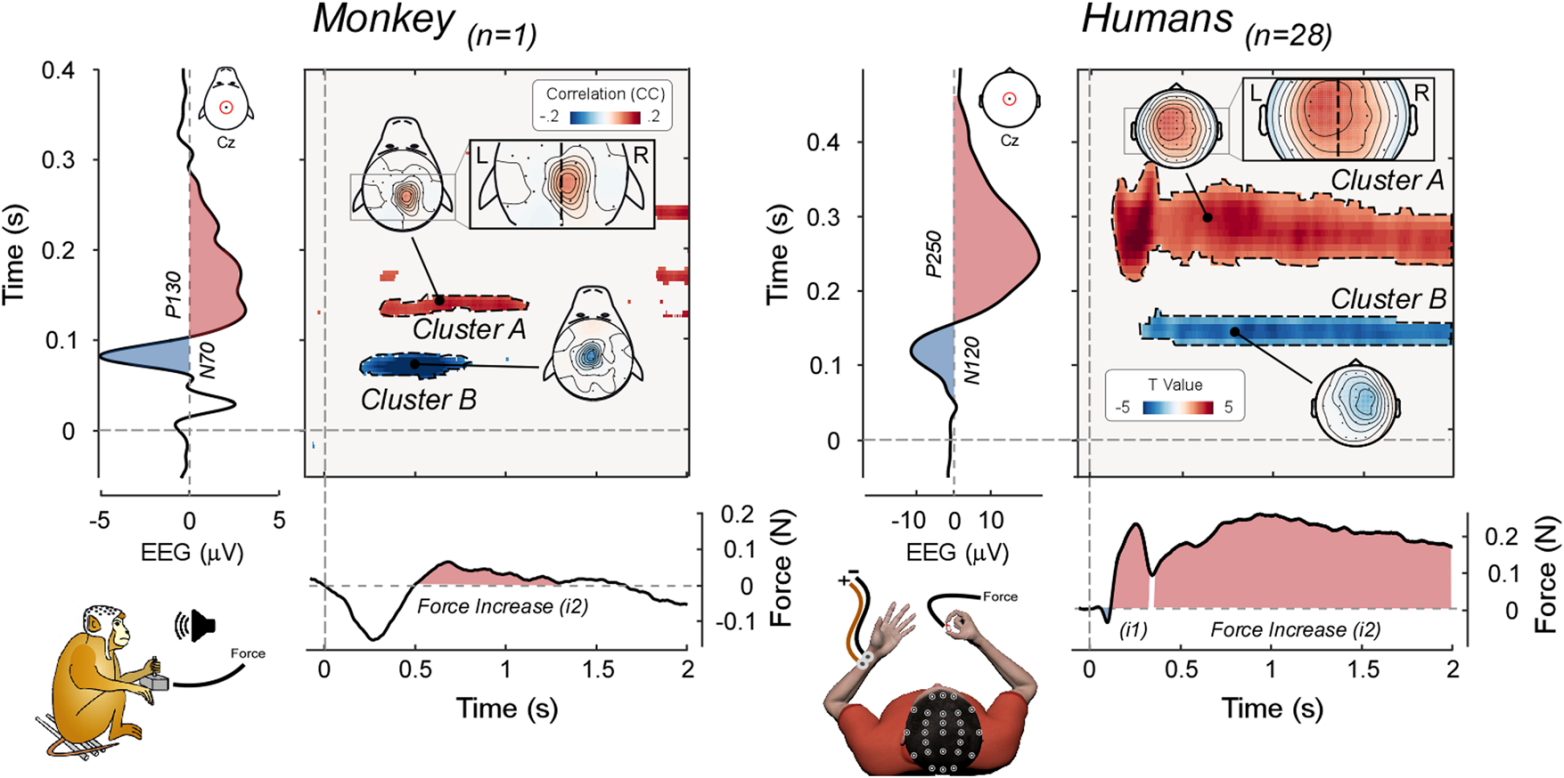
Comparison of stimulus-induced EEG–force correlations in monkeys and humans. Data are from the current study (monkey M, left) and from Novembre et al. (2018; 28 human participants, right). The bidimensional plots represent the significant trial-by-trial correlation coefficients [cluster-corrected Spearman’s *r* (monkey, left), and *t* values obtained by contrasting participants’ Pearson’s *r* values vs. zero (humans, right)] between EEG and force for all possible pairs of time points, at electrode Cz (topographies of the highlighted clusters are plotted). The EEG time series (plotted vertically) and the force time series (plotted horizontally) are shown to assist interpretability of the correlations. Note that the correlation between the positive vertex wave [occurring earlier in monkeys (P130) than in humans (P250)] and the force increase (*i2*) is slightly lateralized toward the scalp regions contralateral to the hand that exerted the force (left hand in monkeys; right hand in human participants). Note that the two datasets were re-referenced differently, likely explaining the more focal (monkey) and global (human) topographies.

In humans, sudden stimuli evoke a complex modulation of constantly applied isometric force (CMR; Novembre et al., 2018, 2019; Somervail et al., 2021; Rangel et al., 2023). An initial force decrease at 100 ms post-stimulus (*d1*) is followed by two force increases: one peaking at 250 ms post-stimulus (*i1*) and the other starting at ∼350 ms and lasting for nearly 2 s (*i2*) (Fig. 7). The current experiments show that monkeys exhibit force modulations reminiscent of the human CMR, with some differences that we discuss in detail. We focus on the force increase, because of its (1) reproducibility across animals and experiments and (2) tight correlation with electrocortical activity.

In both humans and monkeys, salient stimuli evoked a force decrease followed by a force increase. However, while in humans we were able to distinguish two distinct force increases, this was mostly not the case in monkeys (Fig. 7): either they show only one increase (*i2*) or this difference was consequent to holding a joystick using the whole hand (power grip; monkeys) rather than a transducer between the index and the thumb (precision grip; humans; see Fig. 7).

The *i2* observed in monkeys started ∼300–400 ms post-stimulus and lasted 1 s (monkey T) and 1.5 s (monkey M), remarkably similar to the human *i2* [onset, ∼350 ms; duration, ∼2 s (Fig. 7)]. Because of this similarity, we labeled the monkey force increase as *i2*. The human *i1* (onset, 250 ms; duration, 200 ms; Fig. 7) has no homologous modulation in monkeys, at least in the context of the current task.

To study the functional significance of the monkey CMR, we reconstructed the cursor trajectory and made several intriguing observations that might clarify the function of *d1* and *i2* (Fig. 2). The downward cursor drift before stimulus likely reflects the well-known isometric force fatigue (Nazir et al., 2017; Novembre et al., 2018). Therefore, *d1* could be a further transient reduction of the tonic corticospinal output subserving task execution. Similarly, *i2* could be a corrective rebound, bringing the cursor back to its original prestimulus position, but overshooting: cursor position at the end of *i2* (Fig. 2, red dots) is higher than 400 ms before stimulus onset (Fig. 2, black dots). This is consistent with the idea that the CMR is both a reactive and adaptive behavior (RAB; Novembre and Iannetti, 2021).

### EEG/LFP modulations: the vertex potential in rhesus monkeys

Sudden auditory stimuli evoked transient modulations of both EEG and LFP recordings, highly consistent within- and across-animals (Figs. 3–5). An early positivity (P30) was followed by a negativity (N70) and a final positivity (P130), consistent with previous recordings (Neville and Foote, 1984; Pineda et al., 1989; Gil-Da-Costa et al., 2013; Milne et al., 2016).

Sudden auditory stimuli evoke a similar triphasic pattern in humans, although with longer latencies (P50-N100-P200). The latter two components, often labeled N1 and P2, constitute the widely studied “vertex potential,” which indexes “surprise” in response to isolated stimuli regardless of their sensory modality (Bancaud et al., 1953; Mouraux and Iannetti, 2009; Somervail et al., 2021). The EEG/LFP responses in monkeys are highly reminiscent of the human vertex potential, with the shorter latencies explained by the shorter fiber tracts in macaques (Ringo et al., 1994; Caminiti et al., 2009; Woodman, 2012). Notably, despite the coupling with motor behavior (further discussed below), the vertex potential should not be confused with other ERPs classically associated with action preparation such as the lateralized readiness potential (LRP). Indeed, the LRP is constituted by a single monophasic component, with different topography and timescale, besides being elicited in different experimental paradigms (Kornhuber and Deecke, 1965; Vaughan et al., 1968).

Despite the thick muscles surrounding the ears and neck of macaques (Woodman, 2012), we obtained remarkably neat EEG topographies, extremely similar to those observed in humans (Mouraux and Iannetti, 2009; Luck, 2014). By combining well established with recently developed EEG denoising algorithms (independent component analysis and artifact subspace reconstruction (Jung et al., 2000; Kothe and Makeig, 2013; Plechawska-Wojcik et al., 2018), we provide one of the most comprehensive characterizations of event-related potentials in awake monkeys (Fig. 3).

### Neurophysiology of the CMR

The second objective of this study was to investigate the neurophysiology of the CMR. In humans, the CMR modulations are tightly coupled to the electrocortical responses elicited by the same sudden and unexpected stimuli (Novembre et al., 2018, 2019): the trial-by-trial amplitude of both the negative and positive vertex potential waves (N100, P200) strongly predicts the magnitude of CMR force increases. Here we show a similar coupling in monkeys (Fig. 7).

The P130 in EEG/LFP (equivalent to the human P200) was positively trial-by-trial correlated with the force *i2,* in both animals (Figs. 4, 5, clusters A). It is worth noting that while the P130 scalp distribution was symmetrical (Fig. 3*b*), the scalp distribution of this correlation had a hint of lateralization toward the hemisphere contralateral to the hand exerting the force (Fig. 4, insets). This suggestion of a discrepancy between voltage and correlation topographies, together with the clearer dissociation previously observed in humans (Novembre et al., 2018), suggests that corticospinal projections originating in the frontal cortex contralateral to the hand performing the task might be modulated by the vertex potential. This possibility is not conclusive, and we refer to Novembre et al. (2018) for a discussion on the possible existence of a third structure modulating both the vertex potential and the motor cortex producing the CMR. Still, the possibility of a cortical origin of the CMR cannot be ruled out especially when considering that LFPs were measured from the right dorsolateral prefrontal cortex contralateral to the limb performing the task (Figs. 1, 5). Thus, EEG/force and LFP/force correlations in monkeys replicate and extend human observations, providing strong evidence that cortical and muscular responses elicited by sudden and unexpected environmental events are strongly coupled.

Other correlations should be interpreted with caution, as they were inconsistent across animals, although sometimes consistent with human results (Figs. 4, 7, clusters B). Consistently with human results, in monkey M the trial-by-trial amplitude of the N70 (homologous of the human N100) correlated negatively with the *i2* magnitude: a larger N70 predicted a stronger subsequent *i2*. Observing the N70-*i2* correlation in one animal and the P130-*i2* correlation in both animals is consistent with the less robust N100-*i2* correlation (*p *= 0.019) and the stronger P200-*i2* correlation (*p *< 0.001) in humans (Novembre et al., 2018). Unexpectedly, in monkey T the CNV amplitude correlated negatively with the *i2* magnitude. Given that this result was observed only in one animal, and that several equally valid post hoc explanations could be put forward, we prefer to be cautious and report it without providing potentially incorrect interpretations.

Given that we only used correlational techniques, it is difficult to identify the circuits potentially mediating the CMR. We recorded from BA9, a high-order associative region shown to control hand force in both monkeys and humans (Ehrsson et al., 2000; Vaillancourt et al., 2007; Badoud et al., 2017). Unilateral lesioning BAs 9/10 impairs hand force control, leaving other motor behaviors intact. Human studies have shown that this area is part of a network subserving grip force control (Ehrsson et al., 2000, 2001; Vaillancourt et al., 2007; Neely et al., 2013), important for real-time monitoring of force control accuracy, taking into account sensory feedback (Ehrsson et al., 2001; Neely et al., 2013). These observations and our results make BA9 a suitable candidate region mediating the CMR. Notably, other RABs (online motor correction, action stopping) have been associated to dlPFC activity (Cisek, 2007; Scott, 2012; Wessel and Aron, 2017; Novembre and Iannetti, 2021). The role of BA9 might unify these distinct lines of research and suggest a unified neural network mediating the fast modulations of motor output in response to sudden environmental stimuli (Novembre and Iannetti, 2021). Still, whether and through which pathway BA9 might influence the motor output and lead to the observed force modulations remains an open question to be addressed in future studies using causal approaches.

Furthermore, it is important to highlight that recording from a single area limits result interpretability, particularly given that sudden stimuli activate large and widespread cortical territories (Fig. 3; Mouraux and Iannetti, 2009; Liang et al., 2010). We cannot therefore exclude that BA9 does not specifically modulate the motor output and that other cortical areas would show similar LFP responses and correlation with CMR components. Studies entailing multiple intraparenchymal recordings will be necessary to test this likely alternative possibility.
